# Nanoemulsions of Green Tea Catechins and Other Natural Compounds for the Treatment of Urinary Tract Infection: Antibacterial Analysis

**DOI:** 10.15171/apb.2019.047

**Published:** 2019-08-01

**Authors:** Atinderpal Kaur, Reema Gabrani, Shweta Dang

**Affiliations:** Department of Biotechnology, Jaypee Institute of Information Technology, A-10, Noida, U.P., 201309, India.

**Keywords:** Biofilm, Cranberry, Curcumin, Cytotoxicity, Green tea, Nanoemulsion

## Abstract

***Purpose:*** Nanoemulsions (NEs) of polyphenon 60 (P60) and cranberry (NE I) and P60 and curcumin (NE II) were prepared with the aim to enhance anti-bacterial potential and to understand the mechanism of anti-bacterial action of the encapsulated compounds.

***Methods:*** To evaluate the antibacterial potential of the developed NE, microtiter biofilm formation assay was performed. The cytotoxicity analysis was done to assess the toxicity profile of the NEs. Further antibacterial analysis against uropathogenic strains was performed to check the developed NEs were effective against these strains.

***Results:*** In microtiter dish biofilm formation assay, both NE formulations inhibited the growth more effectively (Av. % inhibition ~84%) as compared to corresponding aqueous solution (Av. % inhibition ~64%) and placebo (Av. % inhibition ~59%) at their respective minimum inhibitory concentration (MIC) values. Cytotoxicity analysis using 3-(4,5-Dimethylthiazol-2-yl)- 2,5-diphenyltetrazolium bromide (MTT assay) showed that the formulations were nontoxic to Vero cells. The antibacterial studies against uropathogenic resistant strains also showed that NEs effectively inhibited the growth of bacterial strains.

***Conclusion:*** From different studies it was concluded that both the NE’s were able to inhibit bacterial strains and could be further used for the treatment of urinary tract infection (UTI). The antibacterial activity of developed NEs showed that these could be used as alternative therapies for the treatment of UTI.

## Introduction

Due to the increasing use of antibiotics, resistant strains of bacteria are developing; as a result it is becoming tough to eradicate infections.^1^ The conventional view of antibiotic resistance is one where bacteria exhibit significantly reduced susceptibility to antimicrobials by various mechanisms such as drug inactivation or modification, alteration of target site, alteration of metabolic pathway and reduced drug accumulation by decreasing drug permeability.^2^ Alternative strategies such as use of plant compounds have been employed to preserve the efficacy of existing drugs by preventing development of resistance. Natural compounds have already been used to treat many infections as they are considered to be more safe and reliable and are not exploited commercially. Green tea catechins (P60), obtained from plant *Camellia sinensis*, commonly known as Green tea, are known to have anti-microbial, anti-oxidative, anti-inflammatory, anti-tumour, and anti-aging properties.^3^ It prevents the adhesion of bacterial micro-organisms to the cell wall, hence keeping the infection at bay. But one of the difficulties associated with P60 is that gram negative bacteria are less susceptible to it, as they have an additional lipopolysaccharide layer which prevents the cell lysis.^4^ For this, combinations of different synergistic compounds along with P60 are being investigated to provide better antimicrobial properties. This combinatorial therapy is hypothesized to prevent development of resistant strains and also reduce the risk of dose related toxicity that could be caused due to the accumulation of an individual agent. It was found in our lab that P60 when combined with Cranberry shows synergistic action against *E. coli*.^5^ Similar results were obtained when P60 was combined with Curcumin.^6^ Cranberry extract is obtained from the fruits of plant *Vaccinium macrocarpon*.^7^ It is well known for its antimicrobial action, and especially for the prevention of urinary tract infections (UTIs). Curcumin is a phytochemical present in plant *Curcuma longa* L. rhizome, also known as Turmeric. Curcumin has anti-bacterial, anti-fungal, anti-viral, anti-malarial, anti-inflammatory, and anti-oxidative activities.^8^

But the problem associated with these natural compounds is the low bioavailability, poor solubility, and less stability.^9^ To overcome these issues, the phytochemicals were encapsulated into o/w NEs. It is reported that natural compounds encapsulated in a single NE system help to have an enhanced antibacterial effect using a single carrier.^10^ Many studies have been carried out in which natural compounds were encapsulated in nano carrier systems for the effective delivery of phyto compounds. Nazemiyeh et al prepared stable lycopene-SLNs with good physicochemical characteristic which make it a good alternative for future in nutraceutical industries.^11^ In another study by Eskandani et al, anticancer activity of galbanic acid was improved by loading it in the solid lipid nanoparticles and results confirmed the sustained release of galbanic acid nanoparticles over galbanic acid.^12^ Eskandani and Nazemiyeh also prepared shikonin loaded solid lipid nanoparticles and found that the developed nanoparticles showed improved therapeutic efficacy of shikonin.^13^ Liang et al prepared NE of peppermint oil and proved that NE technology can extend the shelf life of the plant products as compared to their aqueous solutions.^14^ Another study by Moghimi et al, also confirmed that the NE loaded with *Thymus daenensis* appeared to amplify the antibacterial activity of drug against *E. coli* bacteria by enhancing its ability to disrupt cell membrane.^15^ In the present work, o/w NEs were prepared; P60 and cranberry (NE I) and P60 and curcumin (NE II). The objective of the work was to study the effect of phytochemicals encapsulated in NE’s on their anti-microbial potential and cell viability.

## Materials and Methods

### Materials

Polyphenon 60 or green tea catechins (GTCs), curcumin, soybean oil, glycerol and reagent grade Tween 20 was obtained from Sigma Aldrich (Bangalore, Karnataka, India). Cranberry powder was generously gifted by Naturex, DBS, South Hackensack, New Jersey (USA). Trolox was purchased from Calbiochem (unit of Merck Millipore, Vikhroli, Mumbai, Maharashtra, India). Nutrient dehydrated agar and nutrient dehydrated broth were obtained from Qualigens, Mumbai, Maharashtra, India. Polyphenon 60, fetal bovine serum (FBS) and Dulbecco’s Modified Eagle Medium (DMEM) were purchased from Sigma Aldrich, Mumbai, Maharashtra, India. All the other chemicals used in the study were of analytical grade.

*Escherichia coli* (MTCC 739) for preliminary studies was obtained from MTCC, Chandigarh, India. The bacterial culture was maintained in nutrient broth. The isolates for antibacterial studies were maintained on Luria-Bertani (LB) Agar slants and stored under refrigerated conditions.

#### Development of formulations

On the basis of synergistic effect of cranberry and curcumin with Polyphenon 60, two formulations were developed following optimized conditions using homogenization and ultrasonication techniques as in (Table 1).^4,5^ The prepared NEs (P60+Cran NE and P60+Cur NE) were in nano range with particle size of 58 nm and 211.2 nm, respectively. The zeta potential was also within the required range and showed stability of formulations with value of -16 mV for P60+Cran NE and -32.7 mV for P60+Cur NE. The transmission electron microscopy also showed similar size of particles and mono dispersed NEs.^4,5^ These developed formulations were further assessed for different antimicrobial parameters.

**Table 1:** Developed and optimized nanoemulsion formulations

	**Cranberry + P60 NE** **(NE I)**	**Curcumin + P60 NE** **(NE II)**
Oil	Oleic Acid (10%)	Soyabean oil (13.09%)
Surfactant	Tween 20 (20%)	Tween 20 (7.44%)
Co-surfactant	Glycerol (3.52%)	Propylene glycol (8.25%)
Aqueous phase	Water (66.48%)	Water (71.22%)
Drugs	P60 (11 mg/mL)	P60 (16 mg/mL)
	Cranberry (30 mg/mL)	Curcumin (4 mg/mL)
Homogenise	30 min	20 min
Ultrasonicate	300 sec, 30% amplitude	140 sec, 47% amplitude

Abbreviations: NE, nanoemulsion; P60, polyphenon 60.

#### Assessment of antimicrobial activity against Escherichia coli using microtiter dish biofilm formation assay

*Escherichia coli* biofilm was developed by culturing the bacteria on Agar culture.^3^ For the bacterial growth, the biofilm was formed on 96-well microtiter plate by aliquoting 100 mL of the bacterial suspension containing 1 ×10^6^ cfu/mL at 37°C for 48 hours and 100 mL of different concentrations (at 2x, 1x, 0.5x and 0.25x MIC) (MIC (1x) concentration for P60 was 3.3 mg/mL and for Cran was 10 mg/mL and for Cur was 0.3 mg/mL) of NE I and NE II, their placebos and aqueous forms at 37˚C for 48 hours. The culture supernatant was discarded and the plates were washed thoroughly with water after incubation. Subsequently, 100 mL of 0.1% solution of Crystal violet (CV) was used to stain the plate wells and again plates were incubated at 37 °C for 10–15 minutes. The plates were rinsed with distilled water and kept for air drying. To solubilize the absorbed dye 100 mL of 90% ethanol was added. Biofilm formation inhibition was evaluated by taking the optical density at 595 nm of 96-well microtiter plate in an ELISA reader (Biorad, Hercules, CA, USA).^4^ The % inhibition of Biofilm formation was quantitatively determined by using equation 1:

%inhibition of biofilm = 1- [(OD_NE_-OD_negative control_)/ (OD_positive control_-OD_negative control_)] X10 Eq. (1)

#### In vitro cytotoxicity analysis on Vero cell line

Vero cell line was maintained in DMEM medium containing 10% FBS. Vero cells (10^6^ cfu/mL) were seeded in 96-well plate and incubated at 37˚C with 5% CO_2_ for 24 hours to allow the cells to adhere to the plate. The cells were treated with both the NE I and NE II and their aqueous formulations at their MIC (Ix), twice MIC (2x), half MIC (0.5x) and 1/4^th^ MIC (0.25x) values including placebo. After incubation for 24 hours, 20 𝜇L of MTT (3-(4, 5-dimethylthiazol-2- yl)-2, 5-diphenyltetrazolium bromide) prepared in D-PBSA (5 mg/mL) was added to each well and again incubated for 4 hours. The media was replaced by 200 𝜇L of DMSO to terminate the assay.^4^ Absorbance was taken at 570 nm using an ELISA plate reader. The viability (%) was calculated according to equation 2:

%Viability= Af/Ac * 100  Eq. (2)

Where, A*f* = Absorbance obtained for cells treated with formulations, and A*c* = Absorbance obtained for positive control.

#### Adhesion assay

Vero cells grown in DMEM supplemented with 10% heat inactivated FBS were maintained in a 5% CO_2_ environment at 37°C. After attaining confluency, the cells were washed with phosphate buffer saline (PBS), trypsinized, and then counted on a hemocytometer. The cells were seeded on sterile cover slips in a 6 well plate at a concentration of 1 × 10^4^ in complete DMEM. The plate was incubated at 37°C for 24 hours for cell attachment. Media of cells was replaced by secondary culture of *E. coli* at a concentration of 1 × 10^5^cfu/mL in incomplete DMEM containing no antibiotics. In test, Vero cells were incubated with 1 ml of bacterial culture along with 1 mL of aqueous and NE formulations along with placebo of each drug at their MIC values. The plate was incubated at 37°C for 45 minutes. Each well was washed thrice with PBS and then the cells were fixed with methanol and transferred onto glass slides and stained with Giemsa (20% v/v).^3^ Each slide was examined under oil immersion at 100x. Adhesion index was also calculated using equation 3.

Eq. (3)
$$Adhesion\;index=\frac{Total\;mammalian\;cells\;with\;adhered\;bacteria}{Total\;no.of\;mammalian\;cells}*100$$

#### Antimicrobial activity of ESBL and MBL resistant clinical strains by agar well diffusion assay

The effect of NE I and NE II on the test pathogens (*E. coli*, *K. pneumoniae*, *P. mirabilis, C. amalonaticus, C. diversus)* was assayed by agar well diffusion method as per CLSI guidelines. The agar diffusion method was used to determine the minimal bactericidal concentration (MBC) of nano-emulsions. The bacterial culture was diluted to a final concentration of 1 × 10^6^ cfu/mL and was plated on pre prepared Muller-Hinton agar. Different concentrations of NE I and NE II (1:2, 1:5 and 1:10 dilutions) were made and 10 µL was added to each well (0.5 mm) bored on agar plates. Further, the plates were incubated at 37°C for 24 hours and zones of inhibition were measured. MBC was taken as the lowest concentration of nano-emulsion that completely inhibited the growth of pathogens.^16^

## Results

### Assessment of antimicrobial activity against E. coli using microtiter dish biofilm formation assay

The effect of the phytochemicals was tested on *E. coli* biofilms. Biofilm eradication is difficult to achieve due to their inherent resistance to antibiotics. There are several mechanisms explaining the resistance of biofilms to antimicrobials, which makes it difficult to predict the behavior of the biofilm cells.^9^ To investigate the ability of NEs to deliver the drug through bacterial biofilm, the regrowth assay was conducted after incubation, with sub-inhibitory concentration of drug in free form or encapsulated in various NEs. The effect of prepared NE formulations, their corresponding placebos and aqueous solutions on biofilm formation was analyzed via calculation % inhibition at various concentrations (2x, 1x, 0.5x, 0.25x MIC concentrations) (Table 2).

**Table 2:** Antimicrobial activity of developed formulations against *E. coli* biofilm

**Formulations**		**Mean % Inhibition at 0.25X MIC**	**Mean % Inhibition at 0.5X MIC**	**Mean % Inhibition at 01X MIC**	**Mean % Inhibition at 2X MIC**
NE I	P60+Cran NE	73.97	78.37*	82.46	92.62
	Placebo	59.26	69.64	91.04	94.03
	Aqueous Solution	37.37	60.78	80.88	82.95
NE II	P60+Cur NE	56.82	76.50*	96.36*	97.28*
	Placebo	46.34	68.80	85.69	97.03
	Aqueous solution	43.36	48.51	50.13	59.62

Where MIC is the minimum inhibitory concentration; P60 is polyphenon 60; Cran is cranberry; Cur is curcumin; NE is nanoemulsion. * indicated high antibacterial activity of NEs as compared to the aqueous drug solutions. (1x concentration for P60 was 3.3 mg/mL and for Cran was 10 mg/mL and for Cur was 0.3 mg/mL).

In case of NE I, it was observed that % inhibition was 82.46% at MIC (1x) and 92% at twice MIC (2x) concentrations. The corresponding aqueous drug solutions showed a lower %inhibition of 80.88% at MIC (1x) and 59% at twice MIC (2x). However the corresponding placebo showed comparable %inhibition as of NE1 of 91% at MIC (1x) and 94% at twice MIC (2x) concentrations. Similar results were obtained with NE II where formulation showed 96% and 97% inhibition at 1x & 2x MIC respectively. The aqueous solution showed quite lower % inhibition on biofilm formation with only 50.13% and 59.62% inhibition while the corresponding placebo showed 85.69% and 97% inhibition at 1x & 2x MIC concentrations respectively. In both the formulations (NEI & NEII) the %inhibition on biofilm formation for placebo and formulation was observed comparable. The possible mechanism could be because of higher surfactant concentrations present in placebo, killing the bacteria by disrupting the cell membrane. The similar effect was also seen in *in-vitro* cytotoxicity assay where the %cell viability of placebo was around 13%, where the high concentration of surfactants was disrupting the animal cell membrane leading to higher toxicity. On the contrary, the formulations with natural compounds (P60, cranberry and curcumin) the %cell viability values increased drastically by lowering down the surfactant induced toxicity making the prepared formulation comparably less toxic and more effective at the same time. From the observed results, it could be suggested that NEs can be adsorbed on the surface of bacterial biofilm, with subsequent delivery of the encapsulated drug to the bacterial cells. Once again this hypothesis is supported by the superiority of the cationic surfactant (Tween 20), which undergoes strong electrostatic interaction with the negatively charged biofilm and bacterial cell membrane. The enhanced anti-biofilm activity could be attributed due to the synergistic effect of P60+cranberry and P60+curcumin. Our group has earlier confirmed synergistic effect of polyphenon 60 and cranberry in combination against *E. coli* and *S. epidermidis*.^4^ In another study, the MIC of polyphenon 60 and curcumin was determined and it was confirmed that both P60 and curcumin showed synergistic effect with FIC value less than 0.5.^5^ Our results were in agreement with similar studies on the effect of antimicrobial encapsulation in liposomes, the delivery of cationic liposomal benzyl penicillin to a sensitive strain of *S. aureus* immobilized in biofilms was more effective relative to the free drug at low overall drug concentrations and short time of exposure.^17^ Also, as reported by Sanderson and Jones, the encapsulation of vancomycin in cationic liposome enhanced its activity against *S. epidermidis* grown in biofilms, whereas encapsulation in neutral liposomes had less effect than the free drug.^18^ In another study, Borges et al. demonstrated that phenolic compounds such as gallic and ferulic acids reduced the potential of adhesion to some pathogenic bacteria, including *E. coli* and *S. aureus.*^19^

### Cytotoxicity analysis

**Figure 1:**
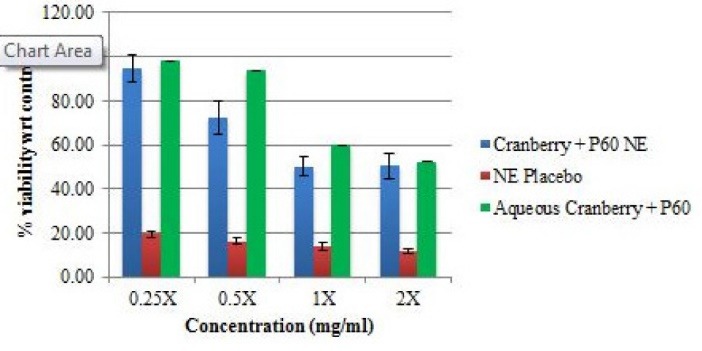
Determination of % viability of Vero cells on treatment with P60 + cranberry NE (NE I), NE placebo and aqueous forms at 0.25X, 0.5X, 1X and 2X MIC values.

**Figure 2:**
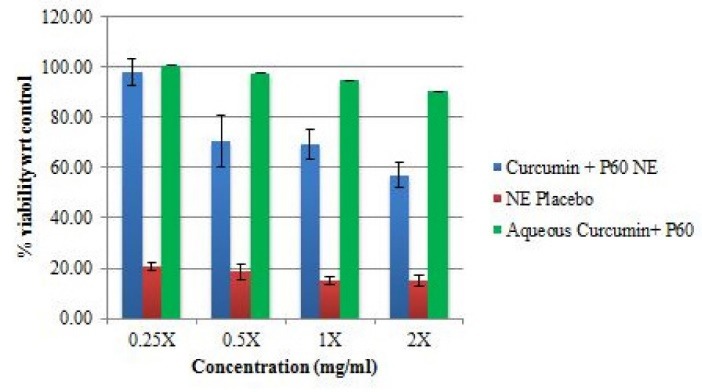
Determination of % Viability of Vero Cells on treatment with P60 + Curcumin NE (NE II), NE placebo and aqueous forms at 0.25X, 0.5X, 1X and 2X MIC values

*In vitro* cell viability of the optimized formulations (NE I and NE II), their corresponding placebos and aqueous formulations was performed on Vero cells (monkey kidney epithelial cell line) using MTT assay. The results of MTT cell viability assay on Vero cell line are shown in Figure 1 and Figure 2. For NE I, it was observed that at the MIC value, the drug loaded NE showed lower %viability (~50%) as compared to the aqueous formulation (~59%). Whereas the placebo showed considerably lower %viability (~13%) at the corresponding MIC value. Similar results were observed in the assay of NE II, the drug loaded NE showed lower %viability (~69%) as compared to the aqueous formulation (~94%) at the MIC value. Whereas the placebo showed very low % viability (~15%) at their corresponding MIC value. This could be attributed to the fact that the surfactant present in the placebo can cause cytotoxicity to the cells. The presence of surfactant tween 20 at high concentration can cause irritation to normal mammalian cells when exposed directly.^20^ The cellular cytotoxicity due to the presence of surfactant in an NE is considerably reduced when natural compounds are encapsulated into them. Thus making the drug loaded NE safe for usage. The results are in agreement with the study by Salvia-Trujillo et al, where it was reported that incorporation of plant products into NEs enhances the antibacterial activity and % viability. In his study, lemongrass loaded NEs were tested for cytotoxicity analysis and it was observed that NE reduced the *E. coli* population up to 0.66, 2.25 and 5.85 log-units after 5, 15 and 30 minutes of contact time.^21^ In our study, in comparison to placebo, the NE formulations were much less cytotoxic on the cells. In another study by Li et al, showed that the surfactants themselves act as cytotoxic to the cells and decreases the %viability of cells.^22^

### Adhesion assay

**Figure 3:**
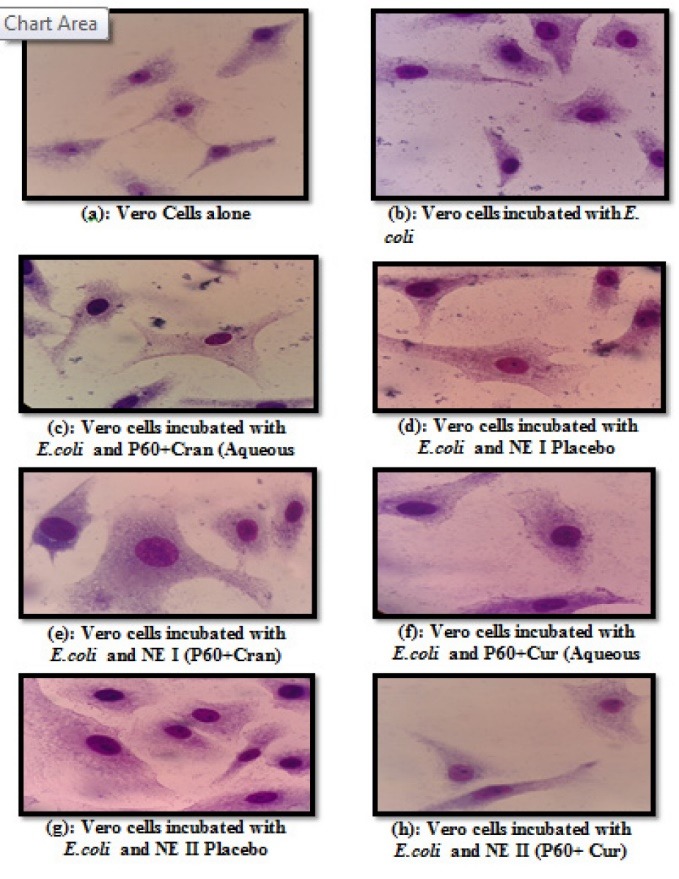
Images of the vero cell lines treated with formulations NE I and NE II at FIC, their Placebo and Aq. forms, Vero cells only and Vero cells treated with *E. coli* (positive control).

The microorganisms have different mechanisms of adhesion and retention, influenced by the substrata, nutrients, ionic strength, pH value and temperature.^23^ Various biomolecules are released by bacteria that assist in attachment to host cells. These could be due to some adhesin molecules that are the cause of interaction between the two cell types. The host cell receptors along with these adhesins are responsible for cellular attachment as they act like bridges between the two cells.^24^ It was observed that the NE I inhibited the adhesion of *E. coli* on Vero cells (Figure 2) at the MIC value with adhesion index of 12.28% for NE, 46.6% for placebo and 24.07% for aqueous solution (Table 3). One possible mechanism of action could be the disruption of the bacterial membrane due the effect of NE. The NE II also inhibited the adhesion of *E. coli* on Vero cells (Figure 3) at the MIC value with adhesion index of 26.53% for NE, 36.36% for placebo and 42.10% for aqueous drug solution (Table 3). Both P60 and Curcumin are effective antimicrobial compounds and the NE considerably lowers down the adhesion index further as compared to the aqueous formulation. Adhesion of a pathogen to the outer surface of host is necessary for its pathogenesis and subsequent infection. Bacterial adhesion to host surfaces starts with the initial attraction of the bacteria to the surface followed by adsorption and attachment, which supports the mechanism of adhesion. During the second phase, specific reactions between substratum surfaces and bacterial surface structures become predominant that causes a firm adhesion of bacteria to a surface via bacterial fimbriae/pili.^25^ A large number of plants compounds have been shown to prevent the adhesion of bacteria to host cell surface. Among them, catechins, cranberry and curcumin has been reported to interact with bacterial outer membrane in a manner that leads to the inhibition of bacterial adhesion to mammalian epithelial cells.^26^ It is to be noted that EGCG, a catechin from green tea would exert a considerable anti-adhesive effect on *E. coli.*^27^ Our results were in agreement with Ramalingam et al study in which the efficacy of a novel NE containing 10% v/v Triton X-100, 25% v/v soybean oil and 1% w/v cetylpyridinium chloride against a multi-drug resistant *A. baumannii* was tested and found that developed NE inhibited the bacterial adhesion to epithelial cell walls.^8^ Another study by Hammer et al, also showed that encapsulation of plant products into a NE increased the hydrophobicity of formulation which results in development of negative charge and inhibition of bacterial adhesion.^28^

**Table 3:** Anti-adhesion Index calculation for developed NE I (P60+Cran); NE II (P60+Cur), their placebos and aqueous forms at MIC values

**Formulations**		**Vero cells with adhered** ***E. coli *** **(%age)**	**Vero cells free from adhered** ***E. coli *** **(%age)**
	Vero cells alone	100%	100%
	Vero cells+ *E.coli*	100%	100%
NE I	*E.coli* infected Vero cells + Aq. (P60+Cran)	24%	76%
	*E.coli* infected Vero cells+ NE Placebo	47%	53%
	*E.coli* infected Vero cells+(P60+Cran) NE	12%	88%
NE II	*E.coli* infected Vero cells + Aq. (P60+Cur)	42%	58%
	*E.coli* infected Vero cells+ NE Placebo	36%	64%
	*E.coli* infected Vero cells+(P60+Cur) NE	27%	73%

Abbreviations: NE, nanoemulsion; P60, polyphenon 60; Cran, cranberry; Cur, curcumin.

### Antibacterial activity against ESBL and MBL resistant clinical strains

**Figure 4:**
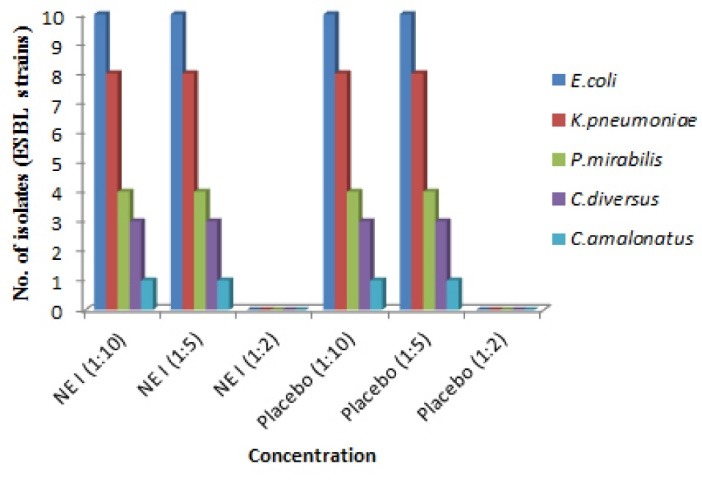
Determination of antibacterial Potential of P60 + Cranberry Nanoemulsion (NE I) against BSL-2 uropathogenic strains (ESBL producing uropathogens). Where 1:2 dilution of both NE and placebo showed growth of bacterial strains and other dilutions showed no growth of strains. (Label claim of Nanoemulsion was 11 mg/mL P60 and 30 mg/mL Cran).

**Figure 5:**
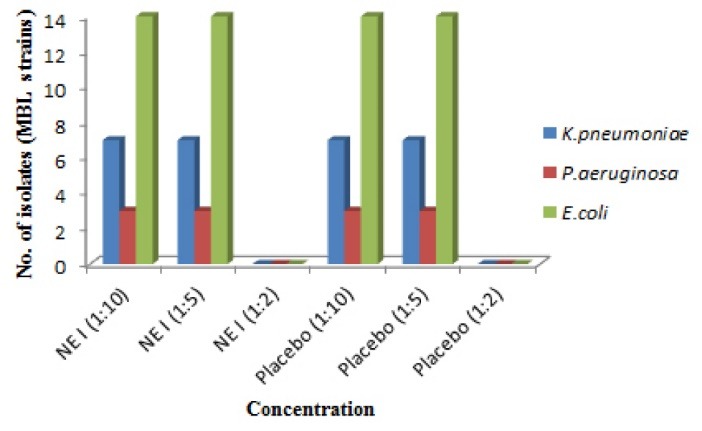
Determination of antibacterial Potential of P60 + Cranberry Nanoemulsion (NE I) against BSL-2 uropathogenic strains (MBL producing uropathogens). Where 1:2 dilution of both NE and placebo showed no growth of bacterial strains and other dilutions showed growth of strains. (Label claim of Nanoemulsion was 11 mg/mL P60 and 30 mg/mL Cran).

**Figure 6:**
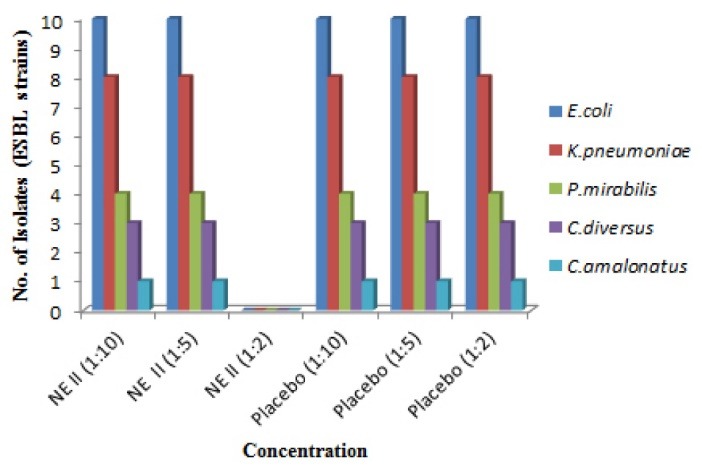
Determination of antibacterial Potential of P60 + Curcumin Nanoemulsion (NE II) against BSL-2 uropathogenic strains (ESBL producing uropathogens). Where 1:2 dilution of NE showed no growth of bacterial strains and other dilutions showed growth of strains. (Label claim of NE was 16 mg/mL of P60 and 4 mg/mL of Cur).

**Figure 7:**
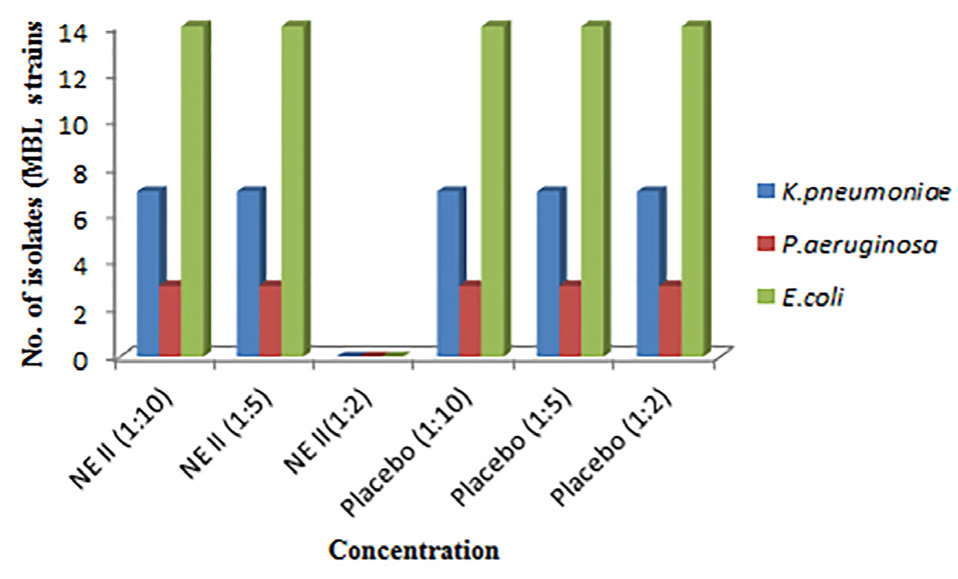
Determination of antibacterial Potential of P60 + Curcumin Nanoemulsion (NE II) against BSL-2 uropathogenic strains (ESBL producing uropathogens). Where 1:2 dilution of NE showed no growth of bacterial strains and other dilutions showed growth of strains. (Label claim of NE was 16 mg/mL of P60 and 4 mg/mL of Cur).

Resistant strains of gram negative bacteria comprised of *E. coli*, *K. pneumoniae*, *Proteus mirabilis, C. amalonaticus, C. diversus*were collected from different health care centers of Mumbai and were identified using conventional cultural, biochemical and morphological tests.^16^ MBC was identified and from the results it was demonstrated that both the formulations showed no inhibition of growth of all the isolates at 1:10 and 1:5 dilution in both extended spectrum beta-lactamases (ESBL) and metallo-beta-lactamase (MBL) producing isolates. Whereas, growth was inhibited at 1:2 dilutions** (**containing 5.5 mg/mL of P60 and 15 mg/mL of CRB; NE I and 8 mg/mL and 2 mg/mL of each P60 and CUR; NE II, respectively). The placebo of NE I showed inhibition of bacterial growth at 1:2 dilutions whereas placebo of NE II formulation showed no growth inhibition of all strains at 1:2, 1:5 and 1:10 dilutions (Figures 4,5,6 and 7). The results indicated that both NE formulations are potent against ESBL and MBL producing uropathogens. Higher antimicrobial effect of NEs can be attributed to the formation of nano drops that increase the surface tension and thereby force themselves to merge with the lipids present in the bacterial cell membrane.^29^ On a mass scale, this effectively disintegrates the membrane and kills the bacteria. Moreover, water present in NE system is tightly bound to the internal oil phase and therefore not available to bacteria for its growth.^30^ Aruna and Mobashshera observed in a study that uropathogens showed higher degree of resistant towards antibiotics.^12^ It also revealed that (72.05%) ESBL producer isolates were resistant to synthetic antibacterial’s, one of the most commonly used first line of treatment for UTI. But our results demonstrated that natural plant compounds encapsulated in a single nano carrier system could be replaced with the synthetic drugs for the effective inhibition of resistant strains and treatment of UTI.

## Conclusion

NEs were prepared in two combinations; P60 and Cranberry (NE I) and P60 and Curcumin (NE II), and these were characterized individually for their antibacterial potential and *in vitro* cytotoxicity on Vero cell lines. The antibacterial study included Microtiter Dish Biofilm Formation Assay, in which it was observed that the NE formulations could inhibit the biofilm formation more effectively as compared to corresponding aqueous formulation at their respective MIC values. The cytotoxicity analysis was done on Vero cells using MTT assay which showed that the optimized formulations were less toxic to Vero cells as compared to the placebo, at their corresponding MIC values. Further, to explore the mechanism of action of antibacterial NEs, adhesion assay was performed, in which Vero cells were simultaneously incubated with *E. coli* and the NE formulations, their placebos and aqueous formulations at their MIC value. It was observed that the NE formulations were more efficient in inhibiting bacterial adhesion to mammalian cells as compared to aqueous formulations. The NE formulations were tested against various resistant strains and findings confirmed the potential of nano formulations to be used against ESBL and MBL producing uropathogens.

## Ethical Issues

There are no ethical issues.

## Conflict of Interest

Authors declare no conflict of interest in this study.

## Funding

This work was supported financially by the Department of Biotechnology, Government of India to conduct the research work (DBT project No. BT/PR7215/NNT/28/654/2013).

## Acknowledgments

The authors are grateful to the Jaypee Institute of Information Technology, Noida, UP (India), for the infrastructural support. The author would also like to thank Dr. Aruna, Wilson College Mumbai for giving facilities and support to carried antibacterial studies.
